# Cartilage Regeneration in Humans with Adipose Tissue-Derived Stem Cells and Adipose Stromal Vascular Fraction Cells: Updated Status

**DOI:** 10.3390/ijms19072146

**Published:** 2018-07-23

**Authors:** Jaewoo Pak, Jung Hun Lee, Natalie Pak, Yoon Pak, Kwang Seung Park, Jeong Ho Jeon, Byeong Chul Jeong, Sang Hee Lee

**Affiliations:** 1 Mipro Medical Clinic, 32-3 Chungdamdong, Gangnamgu, Seoul 06068, Korea; jaewoopak88@gmail.com (J.P.); chxnlxs@gmail.com (N.P.); 2 National Leading Research Laboratory, Department of Biological Sciences, Myongji University, 116 Myongjiro, Yongin, Gyeonggido 17058, Korea; topmanlv@hanmail.net (J.H.L.); ryduses@naver.com (K.S.P.); jeonjh961245@gmail.com (J.H.J.); bcjeong@mju.ac.kr (B.C.J.); 3 First Medical Center, 11841 South St., Cerritos, CA 90703, USA; yoonpak79@gmail.com

**Keywords:** adipose tissue-derived stem cells, stromal vascular fraction, human cartilage regeneration, osteoarthritis

## Abstract

Adipose tissue-derived stem cells (ASCs) in the form of stromal vascular fraction (SVF) and cultured expansion have been applied in clinical settings in some countries to treat osteoarthritis (OA) of knees, one of the most common debilitating, incurable disorders. Since the first report of successful cartilage-like tissue regeneration with autologous adipose SVF containing ASCs, there has been a gradual increase in the number of publications confirming such results. Thus far, most of the reports have been limited to treatments of OA of knees. Recently, successful applications of adipose SVF in treating OA of ankles and hips have been reported. In addition, several groups have reported modified methods of applying adipose SVF, such as combining bone marrow stimulation with adipose SVF or adding additional extracellular matrix (ECM) in treating OA. Here, we present an updated, systematic review of clinical effectiveness and safety in treating OA of knees, ankles, and one hip since 2016 using ASCs in the form of adipose SVF or in cultured expansion, along with a description and suggestion of potential biological mechanisms of cartilage regeneration.

## 1. Introduction

Current medical therapies for degenerative joint disease (DJD) are limited only to symptomatic treatments. Nonsteroidal anti-inflammatory drugs (NSAIDs), hyaluronic acid (HA) joint injections, physical therapy, steroid injections, and even arthroscopic lavage provide only symptomatic relief without addressing the underlying causes of osteoarthritis (OA). Although cartilage regeneration is not the “cure-all” remedy for OA, it can be considered to be a form of curative therapy. When these medical therapies fail, arthroplasty for knee (TKR) or arthroplasty for hip (THR) is the only alternative option of treatment available. However, these surgical measures carry relatively high risks of morbidity and mortality [1,2]. In total, 5.6% of the patients who have received these surgeries experience complications [3,4]. Furthermore, the possibility of adverse outcomes and the finite lifespan of the implanted prostheses necessitating repeated surgical procedures are additional potential limitations of the surgery [5].

Mesenchymal stem cells (MSCs) exist in various human tissues, such as bone marrow and adipose tissue matrix [6,7,8]. These MSCs obtained from adipose tissue matrix are referred to as adipose tissue-derived stem cells (ASCs), which have the capability to differentiate into various tissues originated from the mesoderm, including cartilage [9,10,11]. ASCs have been used in animals and human patients for cartilage regeneration [12,13]. In 2011, Pak, for the first time, successfully treated two human OA patients using a mixture of autologous adipose stromal vascular fraction (SVF) containing ASCs, platelet-rich plasma (PRP), and hyaluronic acid (HA). This mixture was introduced into the diseased knee via percutaneous intra-articular injection [14]. Since then, numerous studies have been published showing similar results [15].

In this review, we present an updated status of the comprehensive and systematic review of publications since 2016 involving the treatment of human OA patients using either autologous adipose SVF cells or culture-expanded ASCs. Also, we will try to ascertain potential biological mechanisms of action of these MSCs in cartilage regeneration.

## 2. ASCs in the Form of Adipose SVF and Cultured Expansion

First, a liposuction needs to be performed to obtain adipose SVF containing ASCs. The adipose tissue procured from the liposuction is referred to as the lipoaspirate. In order to extract ASCs and extracellular matrix (ECM), the lipoaspirate is mixed with collagenase, homogenized, and digested [16,17,18]. Afterwards, the collagenase in the mixture is removed by the dilution method of using normal saline solution and centrifugation in a sterile fashion. After removal of the collagenase, the final volume that is injected into the joint is referred to as adipose SVF, containing several cell and tissue types, including ASCs, ECM, fibroblasts, white blood cells (WBCs), red blood cells (RBCs), and so forth. The ASCs in adipose SVF can further be isolated and culture-expanded [16,17,18]. The process of preparing autologous adipose SVF is considered to be a medical procedure in Korea when it is performed by a physician within a medical facility as a single surgical procedure in the same day with minimal manipulations [19]. On the contrary, culture-expanded stem cells are usually processed in a laboratory and are classified as a pharmaceutical product in Korea [19].

## 3. Potential Biological Mechanisms of Cartilage Regeneration by MSCs

Chondroblasts and chondrocytes are the major cellular components of cartilage tissue, along with the ECM, which makes up the most of the cartilage matrix [20]. The chondroblasts are developed from MSCs, while the ECM is produced by chondroblasts and chondrocytes [20,21]. As chondroblasts mature into chondrocytes, they secrete extracellular matrix, trapping themselves within it. Inside the ECM, chondrocytes further divide into groups of 2–4 cells, forming ECM-covered lacunae [20,21]. The ECM of cartilage is composed of proteoglycan molecules, which are cross-linked and contain fixed negative charges. Proteoglycans, as a component with such a specialized structure, enables the ECM to withstand various different forces [21]. Chondroblasts and chondrocytes in the cartilage tissue maintain the specialized functions of the ECM by regulating synthesis and degradation [20,21].

In OA/DJD, joints become diseased by a variety of factors damaging chondroblasts, chondrocytes, and the ECM, which in turn, causes degradation of the cartilage tissue, resulting in loss of structure and function [22,23]. Such disruption of the tissue is induced by oxidative stress, inflammatory factors, and mitochondrial dysfunction [24]. Mitochondrial dysfunctions have been linked with the pathophysiology of OA/DJD, in which chondrocytes and chondroblasts are found to have reduced mitochondrial functions due to decreased mitochondrial electron transport chain (ETC) proteins [25,26]. ETC proteins are essential for ATP production [25]. Reduction in ETC proteins results in decreased mitochondrial activity, leading to diminished ATP production and thus a decline in the availability of adenosine in the extracellular space [25].

Adenosine in the extracellular space prevents OA phenotypic changes [27]. Extracellular adenosine is derived mainly from the hydrolysis of ATP by the actions of ectoenzymes CD39 and CD73, and mediates its effects via activation of G-protein-coupled receptors (A1R, A2AR, A2BR, and A3R) [27]. Thus, the reduction in ATP leads to decreased availability of adenosine in the extracellular space, resulting in OA phenotypic changes by stimulating expression of matrix metalloproteinases (MMPs), as shown by the following animal study. Mice lacking the A2A adenosine receptor (A2AR) or ecto-5′-nucleotidase, an enzyme that converts extracellular AMP to adenosine, developed spontaneous OA. On the other hand, replacing adenosine by intra-articular injection prevented development of OA [27]. Hence, it can be concluded that the negative factors such as aging, inflammation, and oxidative stress can disrupt the homeostasis of the cartilage matrix and lead to degradation of the cartilage and apoptosis of chondrocytes/chondroblasts, mediated by the lack of adenosine in the extracellular space [28,29,30,31].

MSCs can differentiate into chondroblasts and chondrocytes [21]. In the case of OA/DJD, MSCs can differentiate into chondrocytes, resulting in improvement in joint functions and pain [9,10,11]. Such potential therapeutic function of MSCs can be explained by two possible mechanisms of action: (1) direct adherence and incorporation of MSCs into the host tissue for growth and differentiation and/or (2) trophic effects resulting from the secretome of MSCs. Although the actual true mechanism of action of cartilage regeneration by MSCs is not yet clear, the current evidence is pointing in the direction of both the potential mechanisms working together in harmony [32].

### 3.1. Direct Engraftment

Stem cells have a “homing” effect [33,34,35]. When introduced into a host, stem cells may be able to migrate to the target tissue by interacting with various chemokine receptors, such as CXCR4, integrins, selectins, vascular cell adhesion molecule-1, and so forth [36,37,38,39]. CXCR4, being present on a subpopulation of MSCs, is one of the numerous chemokine receptors involved in MSC migration [36]. Although this is not yet clear, homing is presumed to be significantly dependent on CXCR4 having a binding affinity toward stromal derived factor-1 [36]. Integrins are another family of cell surface molecules associated with cell migration through not-yet-understood pathways. MSCs usually migrate to an infarcted myocardium; however, when integrins are neutralized, the homing of MSCs to the infarcted myocardium is abolished [40]. This is just one example of chemokine receptors being involved in stem cell migration.

After migration via the homing mechanism, MSCs need to attach to and migrate across endothelial cells (ECs) to enter the target tissue. Rüster et al. [37] demonstrated that MSCs, like leukocytes, bind to ECs and migrate by extending podia, followed by rolling and adhesion on the EC. They also showed that the binding and rolling of MSCs were mediated by the P-selectin adhesion molecule, in addition to very late antigen-4 (VLA-4), vascular cell adhesion molecule 1 (VCAM-1), and proteolytic enzymes [37,41].

In 2008, a group in Japan published a report of meniscus cartilage regeneration in rats [42]. The group isolated MSCs from the synovium of the rats, which were inflicted with meniscus damage. Then, the MSCs were introduced into joints of the rats by percutaneous intra-articular injection. After the joint injection, the stem cells migrated to the site of meniscus injury, adhered to the site, and regenerated cartilage, filling the meniscal defect.

In 2017, a group in Korea transplanted umbilical cord-blood-derived (UCB) MSCs along with HA into a rabbit joint to repair articular cartilage defects [43]. They showed that the UCB-MSCs adhered to the site and repaired the defects by regenerating cartilage that had similar cellular architecture and collagen arrangement to the normal cartilage tissue.

These two groups showed that injected MSCs have the ability to attach at the site of damage and repair the host cartilage by regeneration. Furthermore, the first group showed that the MSCs could actually migrate and adhere to the site of damage for tissue regeneration. Although the MSCs introduced definitely attached at the site of injury, the possibility of these MSCs being actually incorporated into the host tissue to transform into the host chondroblasts and/or chondrocytes is not clear.

In the same year, a group in Germany described a “cell tracking system” using a transgenic donor and corresponding immune-competent recipient mouse [32]. Using this method, the group showed that MSCs regenerate cartilage through “non-progenitor” mechanisms [32]. These findings clearly indicated that the adherence of MSC at the site of cartilage defects was necessary; but the attached MSCs just orchestrated the regeneration process instead of transforming themselves into new chondroblasts and chondrocytes in the host tissue.

The above finding was further confirmed by a human clinical trial by de Windt et al. [44]. This group transplanted, via intra-articular injection, allogeneic MSCs and autologous chondrons into knees with cartilage defects. On second-look arthroscopies, the cartilage defects were filled with regenerated cartilage. Biopsies of the regenerated cartilage, however, failed to show any evidence of donor-derived DNA, proving that the transplanted allogeneic MSCs failed to transform into the host chondrocytes or chondroblast. Thus, it can be postulated that the engraftment of stem cells along with the trophic effects produced by MSCs coordinates the regeneration process [32,44].

### 3.2. Trophic Bioactive Factors

MSCs secrete many different bioactive factors that can be categorized into three classes: (1) growth factors, (2) cytokines, and (3) extracellular vesicles [31,45,46,47]. These bioactive factors may have a variety of activities influencing the immune system, the apoptosis, and growth and differentiation of reparative progenitor cells [45,46,48,49]. Extracellular vesicles can be further divided into apoptotic bodies, microvesicles, and exosomes [50].

#### 3.2.1. Cytokines and Growth Factors

MSCs produce a variety of proinflammatory and anti-inflammatory factors. Some examples of anti-inflammatory factors are the hypoxia-inducible factors (HIF), basic fibroblastic growth factor (bFGF), tumor necrosis factor-alpha (TNF-α), transforming growth factor-β1 (TGFβ1), insulin-like growth factors (IGFs), vascular endothelial growth factor (VEGF), interleukin (IL) 13, IL10, IL18 binding protein (IL18BP), IL1 receptor antagonist (IL1RA), anti-apoptotic proteins, and others [51,52,53,54,55,56,57,58,59,60]. Some of the proinflammatory cytokines are IL-1beta (IL1β), IL6, IL8, IL9, and matrix metalloproteinase-3 (MMP-3), among others [53,54,58,59]. Thus, the final anti-inflammatory effects of MSCs are determined by the net effect of these cytokines interacting together. Among these cytokines, hypoxia-inducible factors (HIF) have been reported to promote chondrogenesis [56,60], and insulin-like growth factor-1 (IGF-1) to promote MSC proliferation and differentiation [52,55]. In addition to lowering the amount of inflammatory factors available in the diseased joint, MSCs may prevent the death of chondrocytes by improving the local microenvironment through the expression of antiapoptotic proteins and stimulating the production of inhibitor proteins of apoptosis [51]. Furthermore, MSCs inhibit the production of proapoptotic factors and stimulate the production of antiapoptotic factors [57]. All of these data support the speculation that a variety of growth factors and cytokines produced by MSCs act in concert to promote cartilage tissue regeneration.

#### 3.2.2. Extracellular Vesicles

Extracellular vesicles (EV) are “membrane vesicles that are released by a variety of cells into the extracellular space” and can be “divided into apoptotic bodies, exosomes, and microvesicles” [50,61,62]. When released from stem cells, they may contribute to the regeneration of cartilage via paracrine-like actions. These EVs transfer bioactive cytoplasmic components such as nucleic acids, mitochondria, lipids, and proteins from stem cells to recipient cells [63,64,65,66,67]. Among the subtypes of EVs, most of the available data concern exosomes, showing their significant regenerative properties.

Exosomes are generally referred to as “a specific class of extracellular vesicle characterized by a diameter of 40–150 nm and a density of 1.09–1.18 g/mL” [68]. After being originated from the endosomal system, they are released into the extracellular space [50,69,70]. While in the extracellular space, exosomes are internalized by host cells by fusion with the cell membrane or by phagocytosis, releasing their cytoplasmic contents into the recipient cells, potentially exerting regenerative effects by improving cellular cytoplasmic contents, decreasing death signals, and by immunomodulation [71,72,73].

MSCs are known to produce large amounts of exosomes carrying cargos rich in active glycolytic adenosine triphosphate (ATP)-generating enzymes, along with other cytoplasmic contents [31,46,47]. It is postulated that these enzymes and cytoplasmic contents in exosomes are transferred into the defective cells, for example, chondroblasts and chondrocytes in cartilage, and replenish the reduced mitochondrial ATP production in damaged cells for cellular proliferation and cartilage matrix production.

When cells are injured, ATP is released from the damaged cells into the extracellular space as an immune signal [74]. This extracellular ATP causes immune cells to migrate and accumulate at the site of damage and remove damaged, dying cells [75,76]. This extracellular ATP is hydrolyzed to adenosine monophosphate (AMP), which is converted to adenosine, a potent activator of signals mediated by AKT and ERK pathways [77,78]. The process of degradation of AMP to adenosine is catalyzed by CD73, also known as extracellular ecto-5′-nucleotidase, which is a sure marker of exosomes [79]. Exosomes, through the actions of CD73, may convert extracellular ATP to adenosine.

Adenosine, in turn, activates AKT and ERK signaling pathways, which have been implicated in cellular survival and proliferation [80]. The activated AKT signaling pathway influences many factors involved in apoptosis. In the nucleus, the AKT pathway inhibits transcription factors involved in the expression of cell death genes and enhances the transcription of antiapoptotic genes [81]. In addition, activation of the ERK signaling pathway leads to the phosphorylation of many agents involved in the regulation of cell proliferation. As an example, the ERK pathway is involved in the mitosis phase of the cell cycle by phosphorylating cyclin D complexes [82].

In OA/DJD, immune cells, including macrophages, produce inflammatory cytokines, causing cartilage matrix degradation and joint damage. Macrophages, however, can be further divided into M1 and M2 macrophages [83]. M1 macrophages produce IL6, which inhibits the chondrogenic differentiation of MSCs, while M2 macrophages produce anti-inflammatory IL10, which supports the survival of chondrocytes [22,83,84]. An increase in M2 macrophages was evident in injured immune-competent rats when treated with MSC exosomes [85]. M2 macrophages produce anti-inflammatory cytokines, such as TGF-β1 and IL10, and thus attenuate the effects of inflammatory cytokines such as TNF-α and IL1 [86]. This is an example of the immune-modulating effect of MSCs in cartilage regeneration.

## 4. PRP, HA, and ECM

Some of the studies reviewed in this article utilized either PRP, HA, and/or ECM with adipose SVF or culture-expanded ASCs. The potential rationale for using any one, or more, of these agents is to provide additional complementary effects for ASCs, to achieve better cartilage regeneration by providing scaffold material for stem cells to attach to and/or to stimulate the stem cells to grow and differentiate.

PRP can provide various growth factors which can stimulate the proliferation and differentiation of stem cells [87,88]. In addition to providing a variety of growth factors, PRP may also function like a scaffold material, necessary for stem cells to attach to at the site of cartilage damage after becoming a “curd-like” material by being activated with calcium chloride, thrombin, or collagen [88,89,90,91].

HA and ECM are two naturally occurring scaffold materials. Both HA and ECM have a high affinity for cartilage and provide an environment for stem cells to adhere and attach to the host tissue [92,93]. In addition, ECM secretes a variety of growth factors, which further enhances the stem cells’ growth and differentiation [93].

## 5. Clinical Applications of ASCs in the Form of Adipose SVF and Culture-Expanded Cells

The main features of the clinical studies on ASC therapies for cartilage damage due to OA/DJD published since 2016 are summarized in Table 1.

### 5.1. Retrospective Cohort Study by Kim et al.

This is a retrospective cohort study looking at the short-term result of an adipose SVF injection combined with marrow stimulation while performing supramalleolar osteotomy (SMO) in 64 ankles with varus ankle OA [94]. The clinical outcomes and second-look arthroscopic outcomes of adipose SVF injection with marrow stimulation were superior compared to those of marrow stimulation alone when performing SMO.

As expected, this article shows better results with adipose SVF combined with bone marrow stimulation than bone marrow stimulation alone when performing the SMO surgical procedure. Although this study is interesting, it would have been more worthwhile if the study prospectively compared the effect of adipose SVF alone versus bone marrow stimulation alone, while performing SMO.

### 5.2. Case Series by Fodor and Paulseth

This is a safety and feasibility study of assessing the potential management of OA of eight knees of six human patients with the percutaneous intra-articular injection of autologous adipose SVF obtained by the collagenase digestion of adipose tissue [95]. The knees were injected with, on average, 14.1 million nucleated cells per knee.

After enzymatic digestion of the lipoaspirate with collagenase, on average, 14.1 million viable, nucleated SVF were injected via percutaneous intra-articular injection. Since 1% to 10% of the nucleated cells are ASCs, a maximum of 1.41 million stem cells were injected [17,96]. As shown by Jo et al., potentially a minimum of 10 million ASCs is needed for the joint to achieve adequate cartilage regeneration to be able to be seen in MRI studies [97].

### 5.3. A Phase 1 Dose Escalation Trial by Pers et al.

This is an open phase I clinical trial without a control group. The study was conducted in France and Germany for the evaluation of the safety of a dose-escalation protocol of the intra-articular injection of culture-expanded ASCs in patients with knee OA [98]. There was no correlation with symptom improvement and MRI findings.

This is a dose-escalation study using culture-expanded ASCs. As stem cells go through the culture expansion passages, cells lose the homing effect [34,35]. When injected, some of these stem cells may not migrate to the site of cartilage damage. Also, compared to the study published by Jo et al. in 2014, fewer numbers of stem cells were injected into the knee joint [15]. These two factors: (1) a potentially decreased homing effect and (2) a lower number of stem cells injected may have contributed to the results reported.

### 5.4. Placebo-Controlled Prospective Comparative Study by Nguyen et al.

This is a placebo-controlled randomized study comparing the clinical efficacy of arthroscopic microfracture (AM) with or without the addition of adipose SVF in 30 patients with OA [99].

This comparative study is additional piece of evidence showing the safety and efficacy of adipose SVF joint injections. AM, unlike ASCs, is an invasive procedure that does not regenerate cartilage. Probably, percutaneous injection of adipose SVF without any surgical procedure would be more beneficial for patients if it were to be applied in clinical settings. It would be worthwhile to design a clinical study comparing AM alone versus the percutaneous intra-articular injection of an autologous adipose SVF/PRP mixture.

### 5.5. Case Report by Pak et al.

This case report shows that the percutaneous intra-articular injection of autologous adipose SVF, ECM, HA, and PRP could regenerate cartilage-like tissue in a human hip OA patient [100]. Autologous adipose SVF and ECM were obtained by enzymatically digesting lipoaspirate with collagenase and then homogenizing the mixture. The adipose SVF containing ASCs and ECM was injected into a hip joint along with PRP and HA.

The amount of adipose tissue utilized in this clinical study was about 100 g, which may contain up to 200,000,000 nucleated cells. Of these 200,000,000 nucleated cells, the potential number of ASCs can be 1–10% [17,96]. Thus, a maximum of 20 million ASCs was injected percutaneously into the joint along with ECM and autologous PRP, both of which may release growth factors for stem cells to migrate and attach at the site of cartilage damage [88,89]. HA, being a scaffold material for stem cells, also may have assisted ASCs to regenerate cartilage [92,93].

### 5.6. A Randomized, Double-Blinded Pilot Study by Song et al.

Eighteen patients with OA of knees were randomized into three different groups and received culture-expanded ASCs [101]. The dosage of 5 × 10^7^ ASCs exhibited the highest improvement. The result of this study is consistent with the engraftment and trophic factor theory. When high numbers of MSCs are injected, increased numbers of MSCs can adhere to the site of damage, producing a greater amount of trophic factors for cartilage regeneration.

### 5.7. Retrospective Comparative Study by Kim and Koh

This study looked at the effect of adipose SVF combined with lateral sliding calcaneal osteotomy (LSCO) with bone marrow stimulation [102]. Although the mean VAS (visual analogue scale) and AOFAS (American Orthopaedic Foot & Ankle Society) scores and talar tilt angle radiology improved in both groups, the parameters were significantly better in the group with adipose SVF. ICRS (International Cartilage Repair Society) grades were very well correlated with clinical outcomes in both groups.

Again, as expected, this article shows better results with adipose SVF combined with bone marrow stimulation than bone marrow stimulation alone when performing the LSCO surgical procedure. Although this study is interesting, again, it would have been more worthwhile if the study were prospective, instead of retrospective, and compared the effect of adipose SVF alone versus bone marrow stimulation alone when performing LSCO.

### 5.8. Prospective Cohort Study by Jo et al.

This is a prospective cohort study involving 18 patients with OA of the knees [103]. Although clinical parameters improved for up to two years in all patients, the statistical significance was evident only in the high-dose group. Furthermore, clinical improvement deteriorated after one year in the low- and medium-dose groups, while the improvement reached the plateau in the high-dose group within the two years. The structural outcomes resulted in similar trends.

The result of this study is also consistent with the engraftment and trophic factor theory. When high numbers of MSCs are injected, increased numbers of MSCs can attach at the site of damage, producing a greater amount of trophic factors and regenerating a high volume of cartilage. With greater cartilage regeneration, the improvement of clinical symptoms may have persisted for a longer time duration.

### 5.9. Case Series by Pak et al.

This clinical case series showed that cartilage-like tissue can be regenerated in human knee OA joints by a percutaneous intra-articular injection of a mixture of autologous adipose SVF, ECM, HA, and PRP [18]. Adipose tissue was obtained from the abdominal origin and was minced to extract ECM. The lipoaspirate with ECM was then mixed with collagenase and incubated. The resulting adipose SVF with extra ECM was introduced into the knee joints of three Korean OA patients, along with HA and PRP, via percutaneous intra-articular injection. The knee joints were repeatedly injected with weekly injections of autologous PRP for three weeks. As a result, cartilage-like tissue regeneration was evident in all three patients’ post-treatment MRIs, along with clinical outcome improvements in terms of ROM, VAS, and FRI. This study emphasized the addition of extracted ECM, which was injected with adipose SVF, HA, and autologous PRP. ECM, in addition to HA and PRP, may have enhanced the ability of ASCs to migrate and adhere to the site of cartilage damage.

### 5.10. Randomized, Double-Blind, Placebo-Controlled Study by Kuah et al.

This is a very well-designed study involving 20 knee OA patients with Kellgren–Lawrence (KL) grade 1–3 [104]. The patients were randomized into three groups: (1) a placebo group (*n* = 4), with only cell culture supernatants (CCS) injected, as a control; (2) a 3.9 million ASC group with CCS (*n* = 8); (3) a 6.7 million ASC group with CCS (*n* = 8). All patients received one single intra-articular injection and were followed for 12 months. All patients reported at least one adverse event (AE) after the injection. None were serious AEs, and no withdrawal due to AEs was reported. Statistically significant improvement was noted in terms of VAS in both ASCs groups, while VAS in the placebo group showed marginal improvement. In terms of cartilage regeneration, there was no deterioration in average cartilage volume in the 3.9 million ASC group, while cartilage loss was evident in the placebo group and 6.7 million ASC group. The authors concluded that a single intra-articular injection of ASCs with CCS to patients with symptomatic knee OA was safe.

However, it is difficult to accept the safety claim when 100% of participants experienced adverse events. MSCs are known to have anti-inflammatory effects [59], and numerous human studies, including a safety study reported by Pak et al., did not show 100% adverse events [91]. Thus, the cause of the 100% adverse events should be investigated. Furthermore, the MRI result showed a loss of cartilage volume in the placebo group and 6.5 million ASC group, while no loss of cartilage was evident in the 3.9 million group. It would be interesting to know the exact cause of the adverse events and its potential role in the loss of cartilage volume.

## 6. Discussion

With the accumulation of clinical data, potential mechanisms of action of MSC regeneration of cartilage tissue have been postulated. Although it is not yet clear, the mechanism involves the engraftment of stem cells and their trophic effects working together in harmony. MSCs secrete various bioactive factors: cytokines, growth factors, and extracellular vesicles, which include exosomes that transfer cytoplasmic contents from one cell to other recipient cells. Caplan first postulated that these bioactive factors have trophic effects, regenerating cartilage tissue via autocrine and paracrine fashions [10]. Later, other groups provided evidence that MSCs actually attach at the site of cartilage defects and regenerate cartilage.

In 2017, a German group was able to show that the attached MSCs disappeared after regenerating cartilage [32]. Thus, it can be postulated that after attaching at the site of injury, extracellular vesicles are released and transferred from the donor ASCs to the recipient chondroblasts and chondrocytes.

A safety study reported in 2013 involving the treatment of 91 patients with autologous adipose SVF described a couple of patients repeatedly receiving autologous adipose SVF into the identical knee joints [91]. The group showed that the symptoms of these patients did not correlate well with the number of autologous adipose SVF injections. Such results can be explained by the extracellular vesicle theory. When the second repeated procedure was performed, it can be assumed that there were fewer sites with damage for ASCs to attach. Since fewer cells were attached, fewer extracellular vesicles were available for the host cartilage tissue to regenerate. The result was relatively less improvement compared to the first treatment.

The extracellular vesicle theory may also explain the limited efficacy of the regeneration of cartilage with MSCs. Although the regeneration of cartilage has been documented in various publications, with more stem cells producing better results, none have shown the full amount of growth of cartilage to a normal, undegenerated state. This again can be attributed to the fact that there can only be a limited number of chondroblasts and chondrocytes in the damaged cartilage tissue to regenerate and to produce ECM for cartilage regeneration.

Adipose tissue is an excellent source of MSCs. One gram of adipose tissue may yield up to 2,000,000 nucleated cells, of which 1% to 10% is considered to be ASCs [17,96]. Based on these numbers, we can be certain that a sufficient number of ASCs can be provided to treat OA with an adequate amount of adipose tissue. Since a large number of MSCs attached at the site of injury may produce a huge quantity of trophic factors, it is only logical to assume that utilizing a great number of stem cells would produce better efficacy, as demonstrated by Pers et al., Song et al., and Jo et al. [98,101,103]. In such a sense, culture expansion of the stem cells may be able to produce better efficacy than autologous adipose SVF.

However, stem cells lose their homing effect with a higher number of passages during culture expansions [34,35]. Thus, culture-expanded stem cells with a high number of passages may need a surgical procedure to expose the cartilage lesion for direct application of the stem cells. Adipose SVF stem cells, on the contrary, should have relatively a strong homing effect. Cartilage tissue could be regenerated with percutaneous intra-articular injection of adipose SVF, probably due to the homing effect of stem cells leading them to adhere at the site of cartilage damage.

In addition to introducing a high number of stem cells, growth factors from PRP and ECM may also stimulate stem cells to grow within the joint for better cartilage regeneration. Centeno el al. used autologous platelet lysate to grow bone marrow-derived MSCs in vitro [88]. Pak et al. and other groups have used PRP or another form of platelet-derived materials to enhance stem cell growth in the joint [15,99,100]. Also, ECM and HA have the capability of providing a scaffold material for stem cells to attach at the site of cartilage lesion. Based on the above described presumptive mechanisms of action, MSCs should be able to have positive effects on all other joints of the body, including hips and ankles, as shown by Pak et al. [100] and Kim and Koh [102].

## 7. Method

**Figure 1:**
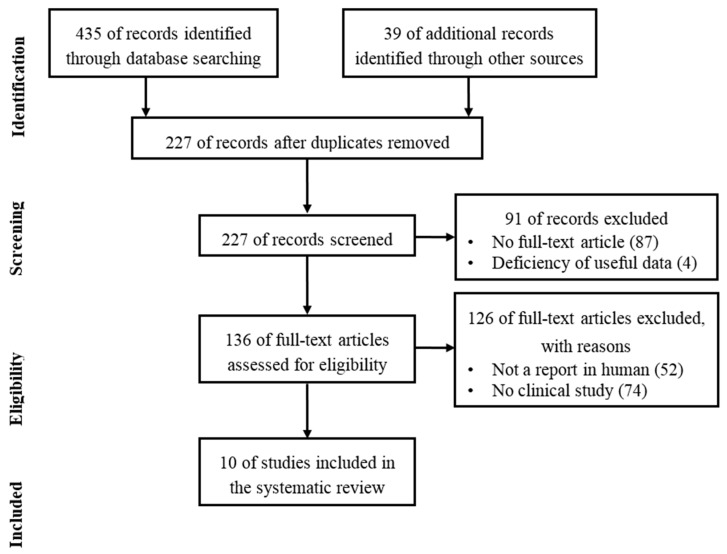
Literature selection process (PRISMA flow diagram).

We used the preferred reporting items for systematic review and meta-analysis (PRISMA) in our review (Figure 1) [105]. We conducted a systematic literature search in the PubMed, Medline, and Embase databases. We used the keywords as our search terms. We combined terms for selected indications (stem cell, osteoarthritis, and adipose). The literature search included all studies published in English between 2016 and 2018. We identified 227 references after removing duplicates. We independently assessed full-text articles for inclusion in our review. The criteria for the inclusion of studies in our review encompassed clinical studies on ASC injection conducted on humans for cartilage regeneration. Finally, we found 10 articles showing clinical studies on ASC treatments for cartilage defects (Figure 1).

## 8. Conclusions

At present, there is no cure for painful OA of knees, hips, and ankles. For these patients, treatment with ASCs, either in the form of adipose SVF cells or culture-expanded cells, can be an alternative option that has been slowly gaining evidence of being safe and efficacious. As data accumulates, the mechanisms of cartilage regeneration by ASCs/MSCs are being elucidated to involve both direct engraftment and trophic factors. Among the trophic factors, extracellular vesicles, especially exosomes, are gaining much attention.

ASC/MSC-based therapy, as with all other cell-based therapies, incurs significant operational efforts and costs as the therapy requires stringent manufacturing processes, storage, and delivery to patients in order to ensure the safety and optimal viability of the cells. Thus, isolating the potential trophic factors responsible for cartilage regeneration may help in overcoming these obstacles and possibly applying the therapy to the general patient population. For now, however, better-designed studies are needed to elucidate the true mechanism of action of the therapy and for the potential general application of these stem cells to treat OA/DJD by cartilage regeneration.

## Figures and Tables

**Table 1:** Clinical studies on treatments with adipose tissue-derived stem cells and adipose stromal vascular fraction cells for cartilage defects.

Study (Year)	Intervention Treatment	Study Type	Number of Subjects/Age (Years)	Subject Characteristic	Concurrent Treatment	Follow-Up	Outcome Measures	Results
Kim et al. (2016) [94]	ASCs harvested from the patient’s buttock ASC injection Arthroscopic marrow stimulation and SMO alone vs. arthroscopic marrow stimulation and SMO + ASCs (4.0 × 10^6^ stem cells)	Retrospective comparative study, level III	62 patients (64 ankles)/51.8: 31 patients/33 ankles Marrow stimulation alone (Group I); 31 patients/31 ankles Marrow stimulation with ASCs injection (Group II)	Varus ankle OA		12.8 months	VAS, AOFAS	The mean VAS and AOFAS scores improved significantly for both groups. There were significant differences in the mean VAS and AOFAS scores between groups at the final follow-up. At second-look arthroscopy, there were significant differences in ICRS grades between groups
Fodor and Paulseth (2016) [95]	ASCs obtained through enzymatic disaggregation of lipoaspirate from the abdomen, flanks, or lateral thighs One intra-articularinjection of ASCs (14.1 million cells)	Case series, level IV	6 patients (8 knees)/59	OA knee		12 months	WOMAC, VAS, ROM, TUG, MRI	Improvement in WOMAC and VAS scores at 3 months and maintained at 1 year. ROM and TUG both improved from preoperative to 3 months. MRI showed no detectable structural differences
Pers et al. (2016) [98]	Autologous ASCs: one intra-articular injection, low dose (2 × 10^6^ cells) vs. medium dose (10 × 10^6^ cells) vs. high dose (50 × 10^6^ cells)	Cohort study, level III	18/64.6: 6 low dose, 6 medium dose, 6 high dose	OA knee		6 months	VAS	Even the low-dose patients group experienced significant improvements in pain levels and function compared with the baseline
Nguyen et al. (2016) [99]	Autologous ASCs harvested from the abdomen isolated arthroscopic microfracture vs. arthroscopic microfracture + ASCs (10^7^ ASCs cells/mL) suspended in PRP	Prospective comparative study, level II	30 patients: 15 patients placebo group/58.2; 15 patients treatment group/58.6	Knee OA (Kellgren–Lawrence grade II–III)	Arthroscopic microfracture and ASC injection	18 months	WOMAC, Lysholm, VAS, Outerbridge classification, MRI	WOMAC, Lysholm, and VAS scores improved; Outerbridge classification, measured with MRI, showed non-differences between the two group, but Outerbridge scores increased in the placebo group over time and decreased in the treatment group
Pak et al. (2017) [100]	Autologous adipose SVF + ECM + PRP + HA	Case report	1 patient	Hip OA		20 weeks	MRI, FRI, ROM, VAS	Along with MRI evidence, FRI, ROM, and VAS all improved
Song et al. (2018) [101]	Autologous culture-expanded ASCs were injected for the low-dose, mid-dose, and high-dose groups, providing three injections and followed up for 96 weeks.	Double-blind, randomized pilot study	18 patients divided into three dose groups: the low-dose (1 × 10^7^), mid-dose (2 × 10^7^), and high-dose group (5 × 10^7^) cells	Knee OA		96 weeks	WOMAC, NRS-11 and SF-36, MRI	Along with MRI evidence, autologous ASCs improved WOMAC, NRS-11, and SF-36 results. The dosage of 5 × 10^7^ adipose MSCs exhibited the highest improvement
Kim and Koh (2016) [102]	ASCs harvested from the patient’s buttock ASCs injection along with arthroscopic marrow stimulation Arthroscopic marrow stimulation vs. ASCs (4.1 × 10^6^ stem cells) + marrow stimulation	Retrospective comparative study, level III	49 patients/53.9: 23 ankles underwent marrow stimulation alone (Group 1), and 26 underwent marrow stimulation with ASC injection (Group 2).	Varus ankle OA		27.6 months 12.5 second-look arthroscopies	VAS, AOFAS, Second-look-arthroscopy	The mean VAS and AOFAS scores improved significantly for both groups. The VAS and AOFAS scores were significantly better in Group 2. Significant differences in ICRS grades between the groups
Jo et al. (2017) [103]	Autologous ASCs isolated from abdominal subcutaneous fat by liposuction and culture-expanded autologous ASCs in normal saline were injected intra-articularly	Cohort study; level of evidence, 3.	18 patients: 3 male/61.8; 15 female/66.6	Knee OA		24 months	WOMAC, KSS, KOOS, VAS, MRI	WOMAC, KSS, KOOS, and VAS improved for up to 2 years regardless of the cell dosage. However, statistical significance was found mainly in the high-dose group. Clinical outcomes tended to deteriorate after 1 year in the low- and medium-dose groups, whereas those in the high-dose group plateaued until 2 years. The structural outcomes evaluated with MRI also showed similar trends.
Pak et al. (2016) [18]	Autologous adipose SVF + ECM	Case series	3 patients: 2 female/60 and 87; 1 male/68	Knee OA		6–22 weeks	MRI, FRI, ROM, VAS	Along with MRI evidence, FRI, ROM, and VAS all improved
Kuah et al. (2018) [104]	Culture-expanded ASCs with culture media supernatant (CMS)	Randomized, double-blind, placebo-controlled Study	20 patients/40–65	Knee OA	None	12 months	MRI, VAS, WOMAC	VAS and WOMAC improved in ASC + CMS groups, but MRI deteriorated in placebo and high-dose ASC + CMS group; no change in low-dose ASC + CMS group

ASC: adipose tissue-derived stem cells; SMO: supramalleolar osteotomy; OA: osteoarthritis; VAS: visual analogue scale; AOFAS: American Orthopaedic Foot & Ankle Society Score; ICRS: International Cartilage Repair Society; WOMAC: Western Ontario and McMaster Universities osteoarthritis index; ROM: range of motion; TUG: time up-and-go; MRI: magnetic resonance imaging; SVF: stromal vascular fraction; ECM: extracellular matrix; PRP: platelet-rich plasma; HA: hyaluronic acid; FRI: functional rate index; NRS-11: numerical pain rating scale; SF-36: short form-36; MSC: mesenchymal stem cell; KSS: Knee Society clinical rating system; KOOS: knee injury and osteoarthritis outcome score.
